# Implementing Intervention Research into Public Policy—the “I^3^-Approach”

**DOI:** 10.1007/s11121-016-0638-3

**Published:** 2016-02-27

**Authors:** Christiane Spiel, Barbara Schober, Dagmar Strohmeier

**Affiliations:** 1 Faculty of Psychology, University of Vienna, Universitaetsstraße 7, 1010 Vienna, Austria; 2 School of Applied Health and Social Sciences, University of Applied Sciences Upper Austria, Linz, Austria

**Keywords:** Intervention research, Implementation science, Public policy, Integrative approach

## Abstract

Evidence-based intervention programs have become highly important in recent years, especially in educational contexts. However, transferring these programs into practice and into the wider field of public policy often fails. As a consequence, the field of implementation research has emerged, several implementation frameworks have been developed, and implementation studies conducted. However, intervention research and implementation research have not yet been connected systematically and different traditions and research groups are involved. Implementation researchers are mostly given mandates by politicians to take on the implementation of already existing interventions. This might be one of the key reasons why there are still many problems in translating programs into widespread community practice. In this paper, we argue for a systematic integration of intervention and implementation research (“I^3^-Approach”) and recommend a six-step procedure (PASCIT). This requires researchers to design and develop intervention programs using a field-oriented and participative approach. In particular, the perspective of policymakers has to be included as well as an analysis of which factors support or hinder evidence-based policy in contrast to opinion-based policy. How this systematic connection between intervention and implementation research can be realized, is illustrated by means of the development and implementation of the ViSC school program, which intends to reduce aggressive behavior and bullying and to foster social and intercultural competencies.

## Implementing Intervention Research into Public Policy

Evidence-based intervention programs in educational contexts have become highly important in recent years. However, transferring these programs into practice and into the wider field of public policy often fails (Fixsen et al. 2013). As a consequence, the field of implementation research has emerged (Rossi and Wright 1984; Ogden and Fixsen 2014). In recent years, a growing body of implementation research has indicated that an active, long-term, multilevel implementation approach is far more effective than passive forms of dissemination (Ogden and Fixsen 2014). Within the field of implementation research, several theoretical bases and models—implementation frameworks—have been developed (Meyers et al. 2012).

However, intervention research and implementation research have not yet been systematically connected and different traditions and research groups are involved. Implementation researchers are mostly given mandates by politicians to take on the implementation of already existing interventions. Moreover, implementation research remains rather isolated and is sometimes considered to be less scientifically valuable than research that develops new interventions (Fixsen et al. 2011). This might be one of the key reasons why there are still many problems in translating programs into widespread community practice (Spoth et al. 2013).

In this paper, we argue for a systematic Integration of Intervention and Implementation research (“I^3^-Approach”). That means researchers design and develop intervention programs based on a field-oriented and participative approach from the very beginning (according to the concept of use-inspired basic research, Stokes 1997; see also Spiel 2009a). This is not only a matter of transferring a program to practitioners at the end of the research process; the whole conceptualization of an intervention as well as its evaluation and implementation should systematically consider the needs of the field (Spiel et al. 2011b) in an integrated way (Beelmann and Karing 2014). Consequently, the perspective of all stakeholders should be included (Shonkoff 2000). Based on theoretical considerations we drew from the literature and our experiences with intervention and implementation research, we summarized the most relevant actions to be taken and issues to be considered on the part of researchers, into a systematic six-step procedure (PASCIT) in order to propose such a systematic connection between intervention research and implementation research. We expect that such a connection would increase the probability of sustainably implementing evidence-based intervention programs into public policy.

How this systematic connection between intervention and implementation research can be realized is illustrated by means of the ViSC Social Competence Program. The main goal of the ViSC program is to reduce aggression and bullying and to foster social and intercultural competencies. It was embedded in a national strategy for violence prevention. For sustainable implementation, a cascaded train-the-trainer (researcher-multipliers-teachers-students) model was developed and applied. Advantages and limitations of the six-step procedure are also discussed for the VISC program as well as from a general point of view at the end of the article.

## Theoretical Background

In recent decades, the evidence-based movement has gained greatly in impact, especially in Anglo-American contexts (Kratochwill and Shernoff 2003). Efforts have been made to make better use of research-based prevention and intervention programs in human service areas such as medicine, employment, child welfare, health, and juvenile justice (Fixsen et al. 2009; Spiel 2009a). As part of this evidence-based movement, various efforts have been made to define standards of evidence. For example, the Society for Prevention Research (Flay et al. 2005) has provided standards to assist practitioners, policy makers, and administrators in determining which interventions are efficacious, which are effective, and which are ready for dissemination (for details, see Flay et al. 2005). Other standards are provided by, for instance, the What Works Clearinghouse (see www.whatworks.ed.gov), the Best Evidence Encyclopedia (see www.bestevidence.org), the Campbell Collaboration (see www.campbellcollaboration.org), and the UK-Based Evidence for Policy and Practice Information and Co-ordinating Centre (see www.eppi.ioe.ac.uk). Common to these standards is the fact that evidence-based programs are defined by the research methodology used to evaluate them, and randomized trials are defined as the gold standard for defining evidence (Fixsen et al. 2009).

However, there are considerable differences in the uptake of research findings among public service areas and scientific disciplines (Nutley et al. 2007). Particularly in the field of education, there has not been extensive implementation of the body of evidence-based research, and the adoption of prevention and intervention programs is driven more by ideology than by evidence (Forman et al. 2013; Slavin 2008; Spiel 2009a, b).

Despite differences among public service fields and countries, it has become obvious that the evidence-based movement has not provided the intended benefits to consumers and communities (Fixsen et al. 2009), and implementing these programs into practice and in the wider range of public policy has often failed (Fixsen et al. 2009, 2013). There is large-scale agreement about one of the central reasons for this disappointment: Program evaluation has not historically included any mention or systematic study of implementation (Meyers et al. 2012). As a consequence, the field of implementation research has emerged (Rossi and Wright 1984). In recent years, a growing body of implementation research has indicated that an active, long-term, multilevel implementation approach (= mission-driven focus) is far more effective than passive forms of dissemination without any active involvement of practitioners (Ogden and Fixsen 2014; Spiel et al. 2012).

Fixsen et al. (2005) defined implementation as the “specific set of activities designed to put into practice an activity or program of known dimensions” (p.5; a similar definition is provided by Forman et al. 2013). Consequently, implementation science has been defined as “the scientific study of methods to promote the systemic uptake of research findings and evidence-based practices into professional practice and public policy” (Forman et al. 2013, p.80; see also Eccles and Mittman 2006).

In the last decade, many implementation studies have been conducted and several conceptual models and implementation frameworks have been presented. The review provided by Meyers et al. (2012) consists of 25 frameworks. They found 14 dimensions that were common to many of these frameworks and grouped them into six areas: (a) assessment strategies, (b) decisions about adaptation, (c) capacity-building strategies, (d) creating a structure for implementation, (e) ongoing implementation support strategies, and (f) improving future applications. According to their synthesis, the implementation process consists of a temporal series of these interrelated steps, which are critical to quality implementation.

Despite these efforts within the field of implementation science, there is agreement among researchers that the empirical support for evidence-based implementation is insufficient (Ogden and Fixsen 2014). Although there is a large body of empirical evidence on the importance of implementation and growing knowledge of the contextual factors that can influence implementation, knowledge of how to increase the likelihood of quality implementation is still needed (Meyers et al. 2012).

Moreover, intervention research and implementation research have not yet been systematically connected. Forman et al. (2013) explicitly pointed out the differences between intervention and implementation activities. While intervention activity refers to the provision of a prevention or intervention program to clients and consists (in the field of school) of a group leader conducting the program with targeted students, implementation activity refers to actions taken in the organizational setting to ensure that the intervention delivered to clients is complete and appropriate. Consequently, different research groups with different research traditions are usually involved in the two tasks. Implementation researchers are mostly given mandates by politicians to take on the implementation of already existing interventions. Moreover, implementation research is seen as less exciting than research that develops new interventions (Fixsen et al. 2011). Furthermore, implementation research is very difficult to do within the constraints of university research environments (e.g., due to time or financial constraints) and is sometimes even considered to be less scientifically valuable (Fixsen et al. 2011; Spiel 2009b).

Therefore, we suggest a systematic integration of intervention and implementation research. Consequently, researchers should design and develop intervention programs using a field-oriented and participative approach (according to the concept of use-inspired basic research, Stokes 1997; see also Spiel 2009a). The whole conceptualization of an intervention as well as its evaluation and implementation should systematically consider the needs of the field (e.g., Spiel et al. 2011a, b, c), and intervention, evaluation, and implementation should be developed in an integrative way. In order to realize this and to avoid as many presumable risks as possible, the perspective of all stakeholders should be included (Shonkoff 2000). In particular, the perspective of policymakers has to be included (Spiel et al. 2011a, b, c; Davies 2004), as well as analyses of what factors support or hinder evidence-based policy (Davies 2004, 2012).

In the next section, we propose an approach for the goal-oriented integration of intervention and implementation research.

## PASCIT—six steps of an “I^3^-Research” (Integrative Intervention and Implementation Research) Approach

Combining theoretical and empirical knowledge from prior research (e.g., Glasgow et al. 1999; Greenhalgh et al. 2004) with our own experience in intervention and implementation research and with the arguments and desiderates described above, we consider at least six steps as constitutive components of an integrative approach to intervention and implementation research. Although these steps mostly occur in succession, some of them might be performed simultaneously. The six steps together must be considered as parts of a dynamic process with many sub-processes, feedback loops, and interdependencies.

### Step 1: Mission-driven Problem recognition (P)

Researchers are used to identify scientific problems and desiderates for new insights. However, in the case of an integrative approach to intervention/prevention and implementation research, the focus is not primarily on problems arising in basic research but on (mostly social) problems in society. Consequently, in order to identify such problems and in order to be generally willing to take action, researchers must not only be curiosity-driven but also mission-driven, combining the quest for fundamental understanding with a consideration of practical use (Stokes 1997). In other words, if scientists intend to contribute to this field of research, the first step requires socio-political responsibility as a basic mindset.

### Step 2: Ensuring Availability of robust knowledge on how to handle the problem (A)

The availability of robust and sound scientific knowledge and evidence is a fundamental precondition for working on an identified problem. Moreover, it is a prerequisite for any kind of transfer (Spiel et al. 2009). Consequently, researchers have to be experts in the relevant field with excellent knowledge of theory, methods, empirical findings, and limitations. This also includes the political dimension of research in the sense of defining and financing corresponding research topics.

### Step 3: Identification of reasonable Starting points for action (S)

The identification of a problem and the availability of relevant insights for initiating changes are not enough if one does not succeed in identifying promising starting points for interventions and their implementation. This must be emphasized, as a wide body of research has made it clear that many intervention programs and measures do not work everywhere and at all times (Meyers et al. 2012). Here again, a necessary condition is high expertise in the relevant scientific field. However, this alone will not do. It must be combined with a differentiated view of prevailing cultural and political conditions. Researchers need knowledge and experience in the relevant practical field and its contextual conditions. This also includes knowledge about potential problems and limitations.

### Step 4: Establishment of a Cooperation process with policymakers (C)

This step is a very crucial one for several reasons. Successful development and implementation of evidence-based intervention in practical settings involves various stakeholders and requires cooperation, persistence, time, and money. In order to conduct integrative intervention and implementation research, stable alliances with the relevant policymakers are necessary, which many researchers traditionally have not established. However, as research often follows its own, very intrinsic logic, which clearly differs from political thinking, a very deliberate process of establishing cooperation and building alliances is necessary. Among other things, this includes more awareness of policymakers’ scope of action. Researchers have to consider that there are other influences on government and policy, beyond evidence. These include values, beliefs, and ideology, which are the driving forces of many political processes (Davies 2004, 2012). Habits and traditions are also important and cannot be changed quickly. Furthermore, researchers’ experience, expertise and judgment influence policymaking, and media; lobbyists and pressure groups also play a role. Researchers have to keep in mind that policymaking is highly embedded in a bureaucratic culture and is forced to respond quickly to everyday contingencies. Last but not least, as resources are limited, policymaking is always a matter of what works at what costs and with what outcomes (Davies 2004, 2012). Consequently, researchers have to find ways to integrate evidence with all these factors. However, again, this step also addresses a certain basic attitude of researchers: It requires that researchers make their voice heard, and sometimes, they have to be very insistent.

### Step 5: Coordinated development of Intervention and Implementation (I)

This step is the centerpiece and a long process rather than a step. The coordinated development has to be performed in a theory-driven, ecological, collaborative, and participatory way. This means that researchers have to include the perspectives of all relevant stakeholders (practitioners, policymakers, government officials, public servants, and communities) in this development process, communicate in the language of these diverse stakeholders, and meet them as equals. Therefore, researchers again have to consider parameters for their research work that differs from many traditional approaches: working together right from the beginning is not common in many fields and also requires new conceptions of e.g., research planning (regarding things like the duration of project phases; see Meyers et al. 2012). Here, the big challenge is to find a balance between wide participation and the maintenance of scientific criteria and standards of evidence, as well as between the freedom of science and research on demand. Consequently, researchers must have theoretical knowledge and practical experience in their very specific field of expertise, but the required profile for a successful “integrative intervention and implementation researcher” is much wider.

### Step 6: Transfer of program implementation (T)

For this final scale-up step, several models and guidelines have been proposed by implementation science (see “Theoretical Background”). Therefore, we will only make a reference to them (e.g., Fixsen et al. 2013; Meyers et al. 2012; Spoth et al. 2013).

In sum, none of the PASCIT steps refer to a completely new consideration or demand (see also Glasgow et al. 1999, who developed the RE-AIM framework for the evaluation of public health interventions), but they have to be seen as one coherent approach. The sound, consistent integration of intervention and implementation research with the goal of introducing changes to policy also requires a (re)differentiation of our scientific identity and the creation of a new, wider job description for researchers in this field.

To illustrate the application of the PASCIT steps of the “I^3^-Approach”, the following sections introduce the ViSC school program, which seeks to reduce aggressive behavior and bullying and to foster social and intercultural competencies.

## The ViSC School Program—Fostering Social and Intercultural Competencies

### Step 1: Mission-driven Problem recognition (P)

Since 1997, violence in schools has gained widespread public attention in Austria, and a number of reports in the media have addressed this topic. As a consequence, a joint statement issued by four federal ministers declared the government’s intention to set up initiatives to prevent violence in several social domains. The government provided financial support for a number of individual intervention and prevention projects in schools. However, as shown in a national report (Atria and Spiel 2003), most of the initiatives taken to prevent violence in schools were organized by individual teachers, researchers were not involved in the planning and organization of these projects. Therefore, these projects and programs were not theoretically based, project goals were imprecisely formulated, and programs were rarely documented and evaluated. In sum, they were a far cry from aligning with the standards of evidence.

### Step 2: Ensuring Availability of robust knowledge on how to handle the problem (A)

Many prevention and intervention programs have been developed by researchers to take on violence at school. They have been evaluated in numerous efficacy and effectiveness trials (e.g., Ferguson et al. 2007; Fox et al. 2012; Smith et al. 2004; Ttofi and Farrington 2009, 2011), and many resources of national institutions have been invested into the implementation of research-based programs in several countries (e.g., Kennedy 1997; Nutley et al. 2007; Petrosino et al. 2001; Shonkoff 2000). It has become apparent that there are at least two key features necessary for program success: (a) to approach the school as a whole and to incorporate a systemic perspective and (b) to conduct activities at school level, class level, and individual level (Smith et al. 2004; Ttofi and Farrington 2009, 2011). However, the deployment of research findings in applied settings has remained slow and incomplete (e.g., Slavin 2002, 2008). It turns out that national strategies actively supported by governments are needed for sustainable violence prevention (Olweus 2004; Roland 2000; Spiel et al. 2011a, b, c).

### Step 3: Identification of reasonable Starting points for action (S)

Consequently, the best means for dealing with violence in Austrian schools was the development of a national strategy with policy and advocacy as important pillars (Roland 2000). Violence prevention programs which take into account the key factors identified for success (Smith et al. 2004; Ttofi and Farrington 2011), comply with the standards of evidence (e.g., Flay et al. 2005) and consider cultural and situational conditions (Datnow 2002, 2005; Shonkoff 2000) should be conceptualized as central parts of this national strategy.

### Step 4: Establishment of a Cooperation process with policymakers (C)

Therefore, we tried to convince officials at the Federal Ministry for Education and the Minister herself of the need for a strategy at a national level by advocating for evidence-based programs and explaining the advantages of a strategic plan as opposed to individual initiatives. Based on several previous projects with the Federal Ministry for Education, we had established an open and honest line of communication.

At the beginning of 2007, in the wake of a quick succession of dramatic, well-publicized incidents in schools and the public discussion of the high rates of bullying and victimization in Austria that followed reports of the results of the *Health Behaviours in School*-*aged Children* (HBSC) survey (Craig and Harel 2004), we received a mandate from the Federal Ministry for Education to develop a national strategy for violence prevention in the Austrian public school system. In preparing the national strategy, we had to cope with the challenge that there was no existing system of collaboration among various stakeholders actively involved in violence prevention and intervention (e.g., school psychologists, social workers, teacher unions) and the lack of knowledge concerning scientific standards. It was therefore our intention to systematically integrate the perspectives of these stakeholder groups in strategy development and to specifically consider the application of theory-based and empirically evaluated prevention and intervention programs (Spiel and Strohmeier 2011).

We developed the strategy between January and November 2007 in a continuous dialogue with officials responsible for this issue at the Federal Ministry of Education and in intensive exchange with international colleagues who had been involved in similar national strategies in their own countries (for details, see Spiel and Strohmeier 2011). In December 2007, the Federal Minister decided to implement the strategy and presented this decision and the strategy plan in a major press conference. For strategy management and implementation, a steering committee was established at the Federal Ministry, with Christiane Spiel as an external member responsible for research issues. In 2008, the national strategy became part of the coalition agreement between the two governing parties and was designed to last up to the end of the legislation period in September 2013. As a consequence, money was devoted to the national strategy and the activities within the strategy. The officers of the Federal Ministry and the Federal Minister herself were very much committed to the national strategy and very keen on getting positive results. The implementation of the strategy continues during the legislation period from 2013 to 2018 (for details about the national strategy and its development, see Spiel and Strohmeier 2011, 2012; Spiel, et al. 2012). As one part of the national strategy, it was possible to expand the so-called ViSC class project (Atria and Spiel 2007), which had previously been developed and applied in rather controlled contexts, into a school-wide program, to develop an implementation strategy for Austrian schools, and to conduct a large-scale evaluation study.

### Step 5: Coordinated development of Intervention and Implementation (I)

In accordance with the national strategy, the main goals of the ViSC program are to reduce aggressive behavior and bullying as well as to foster social and intercultural competencies in schools (Strohmeier et al. 2012). The ViSC program was designed to focus on the school as a whole and to incorporate a systemic perspective. The prevention of aggression and bullying was defined as a comprehensive school development project over the duration of an entire school year. Activities were designed to operate on three different levels: the school as a whole, the classrooms, and the individual (for details, see Spiel and Strohmeier 2011; Strohmeier et al. 2012). In an in-school training for all teachers, basic knowledge on bully-victim behavior and its development was presented. Based on this shared knowledge, the school principal and all of the school’s teachers were encouraged to jointly develop (a) a shared definition of aggression and bullying, (b) shared principles on how to handle aggression and bullying, and (c) commonly agreed-upon measures to sustainably reduce aggression and bullying on the school level. Furthermore, teachers were trained to conduct talks with bullies, victims and their parents in accordance with shared, standardized procedures, in reaction to critical incidents.

One unique feature of the ViSC program concept is that it includes a theory-based project at the class level, which was developed in recognition of the importance of the class context for the prevalence of bullying and victimization. The ViSC class project consists of 13 training units (for details, see Atria and Spiel 2007; Spiel and Strohmeier 2011; Strohmeier et al. 2012). Taking the culture of Austrian schools into account, the ViSC class project is well structured, but open for adaptation in terms of materials and activities used. Before expanding the ViSC class project to the ViSC school program, it had been implemented four times by different researchers in Austrian and German schools. Summative and formative program evaluations confirmed promising results (Atria and Spiel 2007; Gollwitzer et al. 2006, 2007). In particular, a high implementation quality could be found. Out of a total of 52 training units (4 classes × 13 units), only one training unit was deemed not to be in accordance with the project goals. Twenty-nine units were structured exactly according to the training manual and 22 units were in accordance with the project goals, but used adapted materials (Gollwitzer et al. 2006).

The ViSC program’s implementation model was developed concurrently by the same researchers who developed the ViSC program. It needed to take the context and culture of the Austrian school system as well as the concrete situation at each specific school into account (Datnow 2002, 2005). Therefore, and to avoid overburdening teachers and principals, we developed a cascaded train-the-trainer model for the implementation of the ViSC program: researchers train multipliers, multipliers train teachers, and teachers train their students. To train multipliers—known as ViSC coaches—ViSC courses were offered by the program developers. The ViSC courses consisted of three workshops—mostly held at the University of Vienna—and the implementation of the ViSC program in one school. ViSC coaches were required to hold in-school trainings for the entire staff at their assigned school and to supervise and coach them throughout the implementation process. The ViSC coaches also held an additional in-school training for those teachers who planned to conduct the ViSC class project and offered them three supervision units during the implementation of the class project.

### Step 6: Transfer of program implementation (T)

The primary target groups for recruiting ViSC coaches are teachers at educational universities and psychologists. From 2008 to 2014, 55 coaches were trained. To support schools in implementing the ViSC program, many materials were provided on a website, which was presented and explained to teachers by the ViSC coaches. Furthermore, a manual was created to serve as a guide for teachers.

In 2009/10, the ViSC program was intensively evaluated in terms of implementation quality and effectiveness. All secondary schools located in the capital city of Austria were invited to participate in the ViSC program. Out of 155 secondary schools in Vienna, 34 schools applied for participation, out of which 26 schools fulfilled the necessary requirements (participation of the whole school staff in the program, providing time resources for all ViSC trainings, taking part in the evaluation study; Gradinger et al. 2014; Strohmeier et al. 2012). Applying a randomized intervention-control group design, 13 schools were randomly assigned to the intervention group. Out of the remaining 13 schools, only five agreed to serve as control schools. Data from 2042 students (1377 in the intervention group, 665 in the control group) from grades 5 to 7 (47.3 % girls), mean age 11.7 years (*SD* = 0.9), and attending 105 classes were collected at four points in time. In addition, 338 teachers participated in a survey at the pre-testing and post-testing points.

Our first analyses focused on the short-term effectiveness of the program with respect to aggressive behavior and victimization (Strohmeier et al. 2012). A multiple group latent change score model (LCS) to compare the control and intervention group was applied, with gender and age as covariates. The multiple group LCS model, imposing strict measurement invariance, fit the data well. Results indicated a decline in aggressive behavior (the latent mean of the aggression change score in the intervention group, *M* = −0.23, differed significantly from 0; *p* = 0.13), but no change in victimization. Boys scored higher on aggression at time 1 and had lower decreases over time. Age did not have any effects (for details, see Strohmeier et al. 2012). A further analysis using the *Handling Bullying Questionnaire* (HBQ) showed that teachers in the intervention group used more non-punitive strategies to work with bullies and more strategies to support victims compared to teachers in the control group (Strohmeier et al. 2012). We also investigated the short-term effect of the ViSC program on cyberbullying and cybervictimization. Results of a multiple group bivariate LCS model, controlling for traditional aggression, traditional victimization, and age showed effectiveness for both cyberbullying (latent *d* = 0.39) and cybervictimization (latent *d* = 0.29; for details, see Gradinger et al. 2014).

The evaluation of the implementation quality of the ViSC program focused on implementation fidelity (the number of conducted units documented by the ViSC coaches) and participant responsiveness (participation rates of the teaching staff; Schultes et al. 2014). There was a high variability for both scores: implementation fidelity ranged from 0.4 (conduction of 40 % of the prescribed training units) to 2.0 (conduction of twice as many training units as prescribed in the curriculum); the range of participation rates lay between 30 and 100 %. Multilevel analyses showed that teachers’ self-efficacy was significantly more enhanced in schools where the ViSC program had been implemented with high fidelity and that only teachers with high participant responsiveness significantly changed their behavior in bullying situations (for details, see Schultes et al. 2014). We used these results to adapt the training of the ViSC coaches. In a participatory approach together with coaches and school principals, we worked out what conditions are necessary for implementing the ViSC program with high fidelity and high participant responsiveness. In further analyses, implementation fidelity and participation rates will be considered. Currently, the ViSC program is being implemented in Romania and Cyprus by local researchers. Initial evaluations show promising results.

Our intention was not only to implement a program to prevent violence, but also to enable principals and teachers to assess and interpret violence rates in their schools and classrooms as well as to evaluate the effectiveness of interventions against violence without depending on the presence of researchers for data collection, analysis, and interpretation. Therefore, we developed the self-evaluation tool AVEO (Austrian Violence Evaluation Online Tool), which provides information about violence rates from the perspective of both, students and teachers (Schultes et al. 2011; Spiel et al. 2011a, b, c; see also Spiel et al. 2012). The teacher and school perspectives were systematically integrated into the development of the self-evaluation tool and its implementation was carefully evaluated.

## Discussion

Although there is a large amount of empirical evidence for the importance of implementation, there is still not enough knowledge available on how to increase the likelihood of quality implementation (Meyers et al. 2012). From our perspective, one reason for this state of insufficiency might be that intervention and implementation research have not been systematically connected up until now. In this paper, we proposed the systematic integration of intervention and implementation research. We presented an integrative intervention and implementation approach (I^3^-Approach) including a procedure with six consecutive steps (PASCIT) from (1) problem recognition to (6) transfer of program implementation. To illustrate the integration of intervention and implementation, we presented a program from our own research, focusing on violence prevention in schools. In this section, we discuss the strengths and limitations of this example. Finally, problems and challenges of the I^3^-Approach and the PASCIT procedure are examined on a general level.

For the development and implementation of the ViSC school program, it was very helpful that it was part of a national strategy on violence prevention in the public school system, which we ourselves had also developed. Moreover, from our perspective, the establishment of a national strategy was a prerequisite for the upscaling of the ViSC program, as necessary implementation capacity and respective organizational structures (Fixsen et al. 2005) have not been established before. The public discussion of the high rates of bullying in Austria and several incisive events in schools raised policymakers’ awareness of the issue and gave us a window of opportunity for action. A further important step was that the national strategy became part of the coalition agreement of the governmental parties. This solved the budget problem. The national strategy also supported the implementation of sound measures for sustainability (for details, see Spiel and Strohmeier 2012; Spiel et al. 2012) and the realization of a randomized control trial as scientific standard was defined within the strategy. To our knowledge, it was the first time that this gold standard was applied in a program financed by the Austrian Federal Ministry for Education. Further strengths of the ViSC program include the fact that adaptation within a defined range is an explicit part of the program (for details, see Strohmeier et al. 2012); the building of organizational capacity through collaboration with, e.g., school psychology, the ViSC coaches training, the implementation concept and its evaluation, as well as the AVEO self-assessment as a feedback mechanism.

However, there are also some limitations. In the ViSC program, we did not work directly with schools, but rather trained ViSC coaches. This resulted in lower commitment of the schools and lower implementation quality in the evaluation study (Schultes et al. 2014), but advantages for long-term implementation in the school system. Nevertheless, we recommend convincing politicians and government officials that the initial implementation of such programs should be done under the supervision of researchers and program developers. A further limitation has to do with the cultural context. In Austria, responsibility and commitment are not well established in the educational system (see Spiel and Strohmeier 2012). Out of the 155 secondary schools in Vienna invited to participate in the ViSC program, only 34 applied for participation. Considering the high bullying rates in Austria (Craig and Harel 2004), which indicate a need for intervention, the participation rate was low. The low engagement can also be seen by the fact that out of the 13 schools we asked to serve as control schools, only 5 agreed to do so. In other countries, e.g., in Finland (Salmivalli et al. 2013), a greater degree of responsibility in the educational system means that participation rates in such programs are usually much higher.

We tried hard to fulfil all the critical steps identified by Meyers et al. (2012) and were successful concerning most of them (see above), but it was not possible to realize our intention in all cases. To prepare the schools for the intervention, we defined criteria for program participation, such as participation of the entire school staff in the program. The school principals were asked to get consent in advance. However, after the start of the program, it became apparent that this consent had not been obtained in several cases. We further recommended that no other programs should be conducted simultaneously, which was also disregarded by some schools. Finally yet importantly, the school principals’ leadership was not strong enough. This can be attributed to the Austrian school law and cannot be changed easily. School autonomy has been a topic of political discussion in Austria for many years.

Overall, the ViSC program illustrates that intervention and implementation can be systematically combined in the sense of PASCIT. However, it also illustrates that detailed planning of such projects by researchers is difficult and that limitations on different levels—regarding, e.g., cultural contexts—have to be kept in mind.

But why is the systematic combination of intervention and implementation as proposed by PASCIT so difficult? On the surface, the steps seem self-evident. And what is really new in contrast to existing implementation frameworks and transfer concepts? Obviously, most, if not all components (both within and across the six steps) of PASCIT, are already known and have long been considered in intervention and implementation research. However, the new and demanding challenge is our postulation of bringing them together in an integrative and coordinated way, in order to achieve success. The I^3^-Approach represents a very basic but also a very systematic research concept and is more than purely the sum of its steps, ignoring one aspect changes the whole dynamic. RE-AIM (Glasgow et al. 1999) has recommended such a systematic view for the evaluation of public health measures. However, so far, it has not been implemented comprehensively either. Nevertheless, the validity and convenience of PASCIT have to be proven by future research and programs in different fields and cultural contexts.

Obviously, combining intervention and implementation research is very demanding. Therefore, the appropriate acknowledgement in the scientific community is essential. Consequently, individual researchers should not be the only ones engaging in this kind of research; universities also have to include it in their missions. We therefore strongly recommend a discussion of success criteria in academia (Fixsen et al. 2011) and that the social responsibility of academics and universities, respectively, will be considered more deeply. The current gratification system in science is more oriented to basic than to applied research. Within the applied field, it is predominantly technology and medicine that are financially supported and acknowledged by the public. Mission-driven research picking up problems in society is less financed and noticed. Consequently, the number of researchers engaged in this field is limited. However, in the last few years some advances could be observed.

A further problem lies in the availability of knowledge. In the social sciences, particularly in the educational field, it is not easy to get robust scientific knowledge. Reasons for this include that replication studies are rare and only probability conclusions can be drawn. Here, the development of standards of evidence was of high importance (e.g., Flay et al. 2005). However, the requirements defined in these standards are not as comprehensive as demanded by the I^3^-Approach. For example, the evidence standards defined by the Society for Prevention Research (Flay et al. 2005) proposed criteria for efficacious and effective interventions and interventions recognized as ready for broad dissemination. However, they did not combine them with the affordance of implementation.

As mentioned earlier, the commitment of policymakers is crucial. Researchers need to have a great deal of persistence and knowledge about policymakers’ scope of action. However, in most cases this is not enough. A window of opportunity is also needed and researchers have to catch it. Here, the media can be supportive (Spiel and Strohmeier 2012).

To sum up, from our perspective, it is a continuous challenge to introduce the criteria for and the advantages of evidence-based practice to politicians, public officials, and practitioners on the one hand, and to promote the recognition of intervention and implementation research in the scientific community on the other hand. The I^3^-Approach and its PASCIT steps offer one possible procedure for realization. Obviously, other procedures, such as bottom-up approaches, might be possible, especially if implementation capacity (e.g., in the sense of sufficient competent manpower) and respective organizational structures are already established. However, we argue for a systematic, strategic procedure instead of an incidental one.
